# Current status and future opportunities for serial crystallography at MAX IV Laboratory

**DOI:** 10.1107/S1600577520008735

**Published:** 2020-08-21

**Authors:** Anastasya Shilova, Hugo Lebrette, Oskar Aurelius, Jie Nan, Martin Welin, Rebeka Kovacic, Swagatha Ghosh, Cecilia Safari, Ross J. Friel, Mirko Milas, Zdenek Matej, Martin Högbom, Gisela Brändén, Marco Kloos, Robert L. Shoeman, Bruce Doak, Thomas Ursby, Maria Håkansson, Derek T. Logan, Uwe Mueller

**Affiliations:** a MAX IV Laboratory, Lund University, Fotongatan 2, Lund 22484, Sweden; b Department of Biochemistry and Biophysics, Stockholm University, Svante Arrhenius väg 16 C, Stockholm 10691, Sweden; c SARomics Biostructures, Medicon Village, Scheeletorget 1, Lund 22363, Sweden; d Department of Chemistry and Molecular Biology, University of Gothenburg, Gothenburg 40530, Sweden; e School of Information Technology, Halmstad University, Halmstad 30118, Sweden; f European XFEL GmbH, Holzkoppel 4, 22869 Schenefeld, Germany; g Department of Biomolecular Mechanisms, Max Planck Institute for Medical Research, Jahnstrasse 29, 69120 Heidelberg, Germany; h Macromolecular Crystallography Group, Helmholtz-Zentrum Berlin, Albert-Einstein-Strasse 15, 12489 Berlin, Germany

**Keywords:** serial crystallography, macromolecular crystallography, sample delivery, high-viscosity injectors

## Abstract

Over the last decade, serial crystallography, a method to collect complete diffraction datasets from a large number of microcrystals delivered and exposed to an X-ray beam in random orientations at room temperature, has been successfully implemented at X-ray free-electron lasers and synchrotron radiation facility beamlines. This development relies on a growing variety of sample presentation methods, including different fixed target supports, injection methods using gas-dynamic virtual-nozzle injectors and high-viscosity extrusion injectors, and acoustic levitation of droplets, each with unique requirements. In comparison with X-ray free-electron lasers, increased beam time availability makes synchrotron facilities very attractive to perform serial synchrotron X-ray crystallography (SSX) experiments. Within this work, the possibilities to perform SSX at BioMAX, the first macromolecular crystallography beamline at  MAX IV Laboratory in Lund, Sweden, are described, together with case studies from the SSX user program: an implementation of a high-viscosity extrusion injector to perform room temperature serial crystallography at BioMAX using two solid supports – silicon nitride membranes (Silson, UK) and XtalTool (Jena Bioscience, Germany). Future perspectives for the dedicated serial crystallography beamline MicroMAX at MAX IV Laboratory, which will provide parallel and intense micrometre-sized X-ray beams, are discussed.

## Abstract 2

The possibilities to perform serial synchrotron crystallography at BioMAX, the first macromolecular crystallography beamline at MAX IV Laboratory in Lund, Sweden, are described, together with case studies from the synchrotron X-ray crystallography user program.

## 1. Introduction   

### 1.1. Serial synchrotron X-ray crystallography   

Continuous developments in X-ray free-electron lasers (XFELs), more specifically serial femtosecond crystallography (SFX), have enabled data collection from micrometre-sized crystals of several membrane and globular proteins, with decreased risk of radiation damage (Chapman *et al.*, 2014 ▸; Kang *et al.*, 2015 ▸; Liu *et al.*, 2013 ▸; Zhang *et al.*, 2015 ▸; Nass, 2019 ▸). These developments have helped to study molecular dynamics of proteins down to femtosecond time resolution (Tenboer *et al.*, 2014 ▸; Barends *et al.*, 2015 ▸; Nango *et al.*, 2016 ▸; Chapman *et al.*, 2011 ▸) and have succeeded in solving several GPCR structures (Stauch & Cherezov, 2018 ▸). SFX is based on the concept that a complete diffraction dataset can be collected at room temperature from thousands of randomly oriented microcrystals, each exposed to a single very short (5–50 fs) and intense X-ray pulse generated by an XFEL (Martin-Garcia *et al.*, 2016 ▸). SFX beam time is very precious due to the limited number of XFELs and the small number of macromolecular crystallography (MX) stations at each facility. The experiments are often complex and require substantial preparation, including optimization of experimental parameters. In contrast, the number of XFELs cannot be compared with the numerous synchrotron facilities available worldwide. In recent works, data collection using the serial crystallography approach has been performed at third-generation synchrotrons (de la Mora *et al.*, 2020 ▸; Foos *et al.*, 2018 ▸; Owen *et al.*, 2017 ▸; Roedig *et al.*, 2016 ▸; Nogly *et al.*, 2015 ▸). These facilities are more easily accessible for users, available at many more locations around the world and complement experiments at XFELs (Meents *et al.*, 2017 ▸; Oghbaey *et al.*, 2016 ▸). In earlier work, one of the main concerns of performing serial crystallography at synchrotron sources was that radiation damage (Nave & Garman, 2005 ▸) would prevent the collection of diffraction data from microcrystals, since the exposure time would not be short enough to outrun radiation damage, as is the case with femtosecond pulses at XFELs. It was later shown that it is possible to reduce the radiation damage at room temperature also at synchrotron sources by increasing X-ray beam intensity and decreasing sample exposure time (Owen *et al.*, 2012 ▸). Another difference is the peak brilliance of the synchrotrons, which is several orders of magnitude lower than any hard X-ray FEL facility. Nevertheless, serial crystallography at synchrotron beamlines with high flux densities, focused microbeams and fast detectors has been successfully implemented for the development of millisecond (ms) time-scale data collection. Novel developments include different sample delivery techniques (Beyerlein *et al.*, 2017 ▸; Coquelle *et al.*, 2015 ▸; Martin-Garcia *et al.*, 2017 ▸; Nogly *et al.*, 2015 ▸; Owen *et al.*, 2017 ▸; Roedig *et al.*, 2016 ▸; Tsujino & Tomizaki, 2016 ▸) and new data analysis software (White *et al.*, 2012 ▸; Barty *et al.*, 2014 ▸; Sauter *et al.*, 2013 ▸; Kabsch, 2014 ▸). Furthermore, interest in serial synchrotron X-ray crystallography is continually growing within the community, leading to the construction of new micro-focus beamlines dedicated to serial crystallography (*e.g.* ID29, ESRF, Grenoble, France; MicroMAX, MAX IV, Lund Sweden; TREXX, PETRA III, Hamburg, Germany).

### 1.2. MAX IV Laboratory and BioMAX   

**Figure 1:**
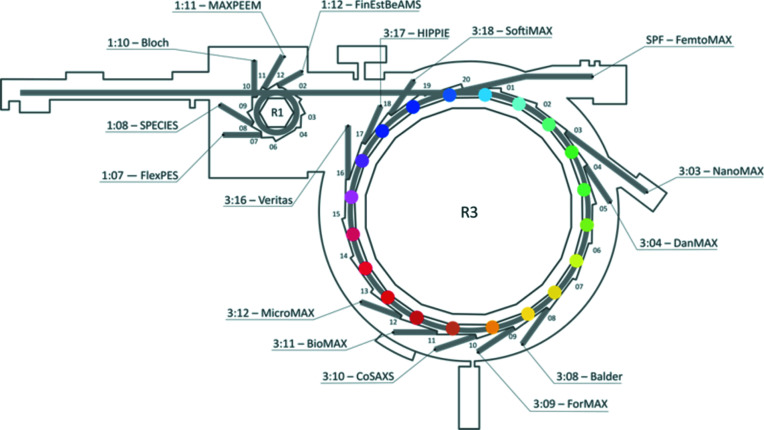
Schematic representation of MAX IV Laboratory photon sources and beamlines (credit: Johnny Kvistholm, MAX IV Laboratory).

MAX IV Laboratory is the first fourth-generation synchrotron storage ring facility in operation worldwide. It utilizes a seven-bend achromat magnet lattice for its 3 GeV storage ring, which dramatically decreases the emittance of the electron beam (Martensson & Eriksson, 2018 ▸) and hence increases the brilliance of the X-ray beam produced. The facility consists of a 3 GeV storage ring, a 1.5 GeV ring and a full-energy linear accelerator (LINAC), which simultaneously functions as the injector for the two rings and for a short-pulse hard X-ray facility (Tavares *et al.*, 2014 ▸) producing 100 fs hard X-ray pulses (Fig. 1 ▸). To date, ten beamlines at MAX IV are open for users (including BioMAX), and six others are currently (July 2020) in commissioning or construction mode.

BioMAX is the first macromolecular X-ray crystallography beamline at MAX IV Laboratory and began user operations in 2017. BioMAX is a 40 m-long energy-tunable beamline, which is fed by an 18 mm period length in-vacuum undulator (Hitachi, Japan). The focused X-ray beam has a cross-section of 20 µm × 5 µm full width at half-maximum (FWHM) at the sample position with a photon flux of 2 × 10^13^ photons s^−1^ at 500 mA ring current. Alternatively, using aperture overfilling, it is possible to obtain a stable 5 µm × 5 µm beam at the sample position which is more suitable for smaller crystals. The energy range of the beamline is 5 keV to 25 keV. The beamline optics consist of an Si (111) double-crystal monochromator and a pair of focusing mirrors in Kirkpatrick–Baez geometry (Ursby *et al.*, 2020 ▸).

The BioMAX experimental setup is suitable for X-ray crystallography with microcrystals and can resolve ultra-large unit cells. The experimental station consists of an MD3 micro-diffractometer (ARINAX, France), an EIGER-16M hybrid pixel detector (DECTRIS, Switzerland) and an ISARA sample changer (IRELEC, France). Data can be collected at 100 K using a Cryojet XL (Oxford Instruments, UK) as well as at room temperature using an HC-Lab humidity controller (ARINAX, France). The EIGER-16M detector can collect full-frame data with a frequency of up to 133 Hz. In the 4M region-of-interest mode, the detector can be operated at up to 750 Hz using only the central eight modules.

## 2. Materials and methods   

### 2.1. Sample environments for SSX at BioMAX   

**Figure 2:**
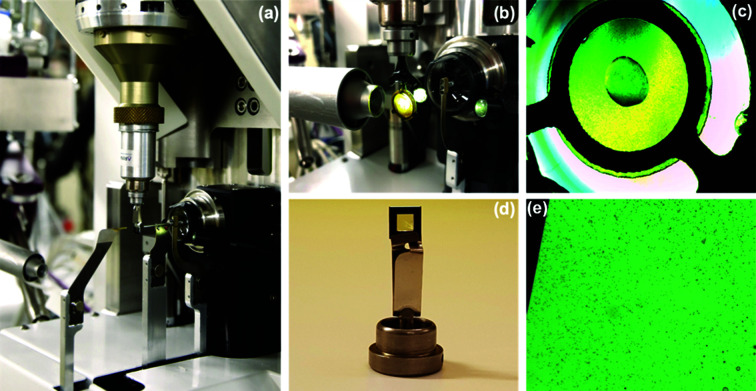
(*a*) SSX raster scan set up at BioMAX. A silicon nitride membrane is mounted on the goniometer head [see panel (*d*]. (*b*) Setup for SSX using XtalTool and a humidifier. (*c*) Crystals grown on a 24-well VDX-plate using XtalTool. (*d*) Omega goniometer head with mounted silicon nitride membrane sandwich. (*e*) 10 µm-sized crystals distributed on silicon nitride membranes.

**Figure 3:**
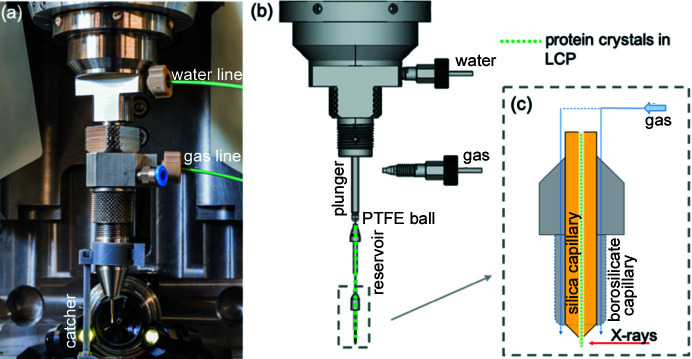
(*a*) HVE injector mounted and prepared for the experiment. The water line is connected to the HPLC pump and is regulated to control the sample extrusion rate. The gas line is connected to a pressurized helium gas cylinder which is controlled for a stable extrusion path. The blue fitting can be optionally connected to the thermostat to maintain a stable sample temperature inside the reservoir. (*b*) Schematic representation of the HVE injector (adapted from the original design by Bruce Doak). The sample shown in green is pushed by a plunger with a PTFE ball at the end through the silica capillary. (*c*) Schematic representation of the end of the injector nozzle. The sample travels through the silica capillary (yellow) and is exposed to the X-rays at the exit. The gas stream travels through the injector down to the borosilicate capillary to stabilize the extrusion.

At BioMAX, room-temperature SSX data have been collected using either the high-viscosity extrusion (HVE) injector or different fixed-target supports (which can be combined with the HC-Lab humidity controller). The MD3-goniometer is equipped with a high-resolution on-axis microscope and sub-micrometre *x*, *y*, *z* sample-centering stage. The standard omega-goniometer head is used to perform fixed-target SSX experiments [Fig. 2 ▸(*a*)]. This device can be easily exchanged with an HVE injector head, which is fully supported by the MD3 environment [Fig. 3 ▸(*a*)].

Fixed-target supports are translated quickly and accurately through the X-ray beam using U-turn raster-scan movements performed by the motion system of the MD3. The fixed-target approach helps to reduce sample consumption, which is particularly useful for proteins that cannot be crystallized in large quantities. Currently, BioMAX uses two different types of fixed-target supports: silicon nitride membranes (Silson, UK), with an area of either 1.5 mm × 1.5 mm or 2.5 mm × 2.5 mm and a thickness of 500 nm, which are clipped onto the goniometer base (Molecular Dimensions, UK) [Figs. 2 ▸(*a*) and 2(*d*)], and a novel solid support, XtalTool [Figs. 2 ▸(*b*) and 2(*c*)], was also implemented and tested at the beamline (Feiler *et al.*, 2019 ▸; Jena Bioscience, Germany). The 21 µm-thick polyimide foil features 5 µm pores, allows direct crystal growth using 24-well plates [Fig. 2 ▸(*c*)] and facilitates direct mounting onto the beamline with a unique goniometer base [Fig. 2 ▸(*b*)]. Both membrane and XtalTool supports are made from highly X-ray transparent materials (silicon nitride and bio-inert polyimide, respectively) which minimize background scattering.

Data collection parameters of the scan are set using the *MXCuBE3* web application (Mueller *et al.*, 2017 ▸). Data are collected with a small degree of rotation for each line (∼1–10°) specified by the user via *MXCuBE3*. Both supports can be used to collect up to 40 000 raster points within minutes. Recently, the MD3 received a performance upgrade to further decrease the data collection time of the scan rate to 60 points per second.

Another way to perform SSX experiments at BioMAX is to use an HVE injector (Fig. 3 ▸) which already produces rewarding results at other synchrotrons (Nogly *et al.*, 2015 ▸; Botha *et al.*, 2015 ▸; Martin-Garcia *et al.*, 2017 ▸). The HVE injector at BioMAX was designed and fabricated at Max Planck Institute for Medical Research (Heidelberg, Germany) to be fully compatible with the environment of the BioMAX experimental station and to enable a long extrusion time, which is permitted by the relatively large working sample volume (Doak *et al.*, 2020 ▸).

The injector body is stainless steel with a maximum sample reservoir volume of 130 µl available for extrusion. This sample volume is sufficiently large to run for several hours, depending on the flow rate, which facilitates the collection of a high number of diffraction images in a continuous fashion. The extrusion pressure is generated by the hydraulic pressure produced by a high-performance liquid chromatography (HPLC) pump (Shimadzu, Japan, Solvent Delivery Unit LC-20AD), which forces water to push against a piston/plunger inside the injector [Fig. 3 ▸(*b*)] at a constant flow rate and pressure. The plunger has a larger diameter on the upper side (water) and a smaller diameter on the sample side, acting as a force multiplier for extrusion of the more viscous sample material. This piston directly pushes a polytetra­fluoro­ethyl­ene (PTFE) ball which acts as a secondary plunger for separation and sealing of the water from the crystal sample [Fig. 3 ▸(*b*)]. The ball continuously pushes the sample from the reservoir into a thin silica capillary [Fig. 3 ▸(*c*)]. The internal size of the silica capillary varies from 50 µm to 100 µm in diameter, depending on the crystal size expected to be used. The sample-loading procedure uses Hamilton syringes with volumes of 100 µl or 250 µl, as described in the work by Botha *et al.* (2015 ▸). At the end of the nozzle, helium gas at a maximum pressure of 50 psi flows through a borosilicate sheath capillary [Fig. 3 ▸(*c*)], which creates a laminar stabilizing sheath gas flow of the extruded crystal sample. Both water flow and He gas flow are controlled remotely to obtain a constant stable flow of sample from the HVE injector. To avoid contamination of the apertures and the surrounding experimental environment, a custom-made sample catcher was designed and 3D-printed. Data collection is started from the *MXCuBE3* experimental control software and the crystal hit rate is calculated on the fly.

### 2.2. Preparation of microcrystals   

Three proteins from three different user groups were crystallized in their home laboratories and afterwards collected at the BioMAX beamline as part of the collaborations for the development of SSX at BioMAX: the C-terminal carbohydrate recognition domain of galectin-3 (galectin-3C, provided by SARomics Biostructures), the ribonucleotide reductase R2 subunit from *Saccharopolyspora erythraea* (hereafter referred to as R2, from the Martin Högbom group at Stockholm University) and *ba*_3_-type cytochrome *c* oxidase (CcO) from *Thermus thermophilus* (a membrane protein from the Gisela Brändén group at University of Gothenburg).

Galectin-3C crystals were grown directly on an XtalTool support (Jena Bioscience, Jena, Germany) using a 24-well VDX-plate with 1 ml reservoir volume [Fig. 2 ▸(*c*)]. The drop was set up using 1 µl 20 mg ml^−1^ protein in buffer containing 10 m*M* sodium phosphate pH 7.4, 100 m*M* NaCl, 10 m*M* β-mercapto­ethanol, 2 m*M* lactose and 0.25 µl of galectin-3C seed crystals in a stabilization solution containing 0.1 *M* Tris pH 7.5, 33% (*w*/*v*) PEG 4000, 0.1 *M* MgCl_2_, 0.2 *M* NaSCN and 0.75 µl reservoir solution containing 0.1 *M* Tris pH 7.5, 30% PEG 4000, 0.05 *M* MgCl_2_, 0.2 *M* NaSCN and 8 m*M* β-mercapto­ethanol. Crystals used for the silicon nitride membrane experiment were grown on the same XtalTool support and VDX-plate using the same protein and seed solution with a slightly different reservoir composition [0.1 *M* Tris pH 7.5, 34% (*w*/*v*) PEG 4000, 0.2 *M* NaSCN and 8 m*M* β-mercapto­ethanol]. Crystals were grown at 4°C to a size of approximately 10–15 µm.

The R2 protein was crystallized at a concentration of 20 mg ml^−1^ using the batch method with a crystallization buffer of 16% (*w*/*v*) polyethyl­ene glycol (PEG) 3350 and 2% (*v*/*v*) tacsimate pH 4.5 in a 1:1 protein solution to crystallization buffer ratio (Lebrette *et al.*, in preparation). Crystals appeared overnight at 21°C in the size range 10–40 µm [Fig. 2 ▸(*e*)].

The membrane protein *ba*_3_-type cytochrome *c* oxidase from *Thermus thermophilus* was purified and crystallized at the University of Gothenburg as described in the work by Anderson *et al.* (2017 ▸, 2019 ▸). The lipidic cubic phase (LCP)-grown crystals 5–20 µm in size were used for data collection at BioMAX.

### 2.3. Data collection and data processing at BioMAX   

Data analysis is one of the major challenges for serial crystallography since each diffraction image contains reflections measured with unknown partialities (Kirian *et al.*, 2009 ▸). In addition, SSX data collection typically generates several terabytes of data, including many frames that contain no usable data and thus should be excluded to speed up the data processing. At BioMAX, initial hit identification from raw HDF5 files can be performed using *Cheetah* (Barty *et al.*, 2014 ▸) or *NanoPeakCell* (Coquelle *et al.*, 2015 ▸). Sorted diffraction patterns can then be indexed, integrated and merged using *CrystFEL* (White *et al.*, 2012 ▸, 2016 ▸). In this article, all data were processed using *NanoPeakCell* and *CrystFEL 0.7.0*. Data collection and processing results are provided in Table 1 ▸. The resolution cutoff was determined based on conservative criteria for a signal-to-noise ratio >2, completeness (100%) and *R*_split_ (<60%) for the highest resolution shell. Data were converted to MTZ format and phased by molecular replacement using *PHENIX* (Adams *et al.*, 2010 ▸) and the structure models from the Protein Data Bank [PDB entries 6eym for galectin (Manzoni *et al.*, 2018 ▸) and 3s8g for *ba*_3_-type CcO (Tiefenbrunn *et al.*, 2011 ▸)]. A structure model for the R2 protein was an unpublished result of a previous data collection (Lebrette *et al.*, in preparation). Model building was performed using *Coot* (Emsley & Cowtan, 2004 ▸). Structures were refined using *phenix.refine* (Adams *et al.*, 2010 ▸).

All data collections were performed at a wavelength of 0.98 Å (12.7 keV) with a photon flux of 2 × 10^12^ photons s^−1^ at 100 Hz frame rate, with a 20 µm × 5 µm beam size. The energy of the beamline monochromator was calibrated using absorption edges (Cu, Se, Zr). Tests using further absorption edges and powder diffraction measurements confirmed that the Bragg axis encoder values are accurate. The beam centre and detector distance were calibrated with LaB6 by PyFAI (Ashiotis *et al.*, 2015 ▸). The average dose per crystal was calculated using *RADDOSE-3D* (Bury *et al.*, 2017 ▸).

The data collection using the HVE-injector took several hours to obtain a complete dataset due to the low hit rate of 4.7% and some beamline issues. Data collections using fixed-target supports were performed with a small degree of rotation (5°) for each support and took less than 1 h to collect a full dataset, including mounting of the samples at the beamline. The average hit rate was 64%.

## 3. Results   

### 3.1. Galectin using XtalTool and silicon nitride membranes   

**Figure 4:**
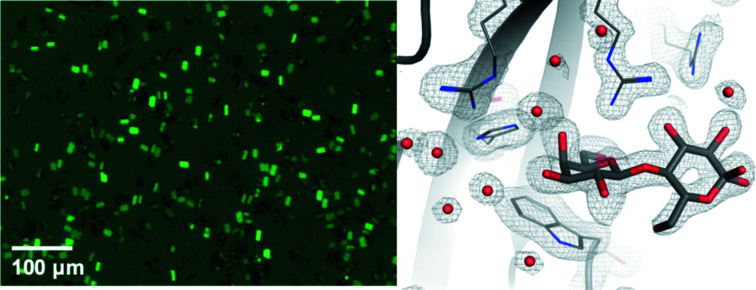
Left: microcrystals of galectin. Right: 2*mF*_o_ − *DF*_c_ simulated annealing omit map, omitting only the lactose residue contoured at 1σ.

SSX diffraction datasets from galectin-3C crystals were collected using XtalTool supports and silicon nitride membranes with U-turn continuous scanning and an average data collection speed of 100 µm s^−1^. The crystal size did not exceed 20 µm (Fig. 4 ▸, left) and the X-ray exposure time per image was 0.05 s for both datasets.

For the dataset obtained with XtalTool, an HC-Lab humidifier was set up at 98.5% relative humidity [Fig. 2 ▸(*b*)]. Full dataset collection required two XtalTool supports, with 2 µl droplets on each. In total, 70 537 diffraction images were collected with a 70.1% hit rate, from which 15 555 could be indexed in the space group *P*2_1_2_1_2_1_ (PDB entry 6y4c). The final structure was refined to 1.7 Å resolution with *R*_work_ and *R*_free_ values of 17.2% and 20.1%, respectively (Table 1 ▸). The resulting experimental electron density maps were of excellent quality, revealing the presence of lactose (ligand). Fig. 4 ▸ (right) demonstrates the high quality of the 2*mF*_o_ − *DF*_c_ electron density map near the ligand.

The structure is very similar to the room temperature complex determined by joint neutron/X-ray crystallography (1.7 Å for neutrons, 1.1 Å for X-rays; PDB entry 6eym). The root-mean-square deviation (r.m.s.d.) in 120 matched Cα positions between the two structures is 0.47 Å, which is higher than the usual r.m.s.d. (<0.2 Å) between galectin-3C structures determined at 100 K. The core of the structure appears most similar, while shifts of up to 0.77 Å are seen in surface loops. The reason for this is unknown but it could be coupled to a systematic reduction in cell dimensions in both SSX datasets, compared with the values usually observed for galectin-3C crystals at room temperature (*a* = 37.2 Å, *b* = 58.5 Å, *c* = 64.0 Å).

The second dataset from galectin-3C crystals was collected using silicon nitride membranes. A volume of 0.4 µl of the sample was distributed directly onto the membrane and covered with a second membrane to create a so-called sandwich. No additional sealing was needed to maintain the humidity of the sample as it can be kept at the beamline for up to 30 min before crystal dehydration at the chip edges can be observed. Subsequently, the sample was clipped onto the goniometer base and mounted at the beamline [Fig. 2 ▸(*d*)]. Two sandwiches were needed to obtain a complete dataset with a total volume of 0.8 µl. The final structure was refined to 1.7 Å resolution with *R*_work_ and *R*_free_ values of 19.2% and 20.8%, respectively (PDB entry 6y78). The results of data collection and data processing are presented in Table 1 ▸. On the whole, the data collected from XtalTool were of slightly higher quality, allowing, for example, the identification of more well ordered water molecules (117 versus 72) at the same resolution.

### 3.2. R2 protein crystals on silicon nitride membranes   

**Figure 5:**
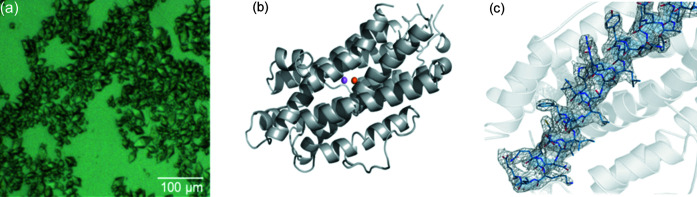
(*a*) R2 protein microcrystals. (*b*) Overall structure of the R2 protein. (*c*) 2*m*|*F*_o_| − *D*|*F*_c_| electron density map contoured at 1σ near one of the α-helices in the R2 structure.

Data were collected using U-turn raster-scanning applied to silicon nitride membranes. The sample preparation and deposition onto the membranes were the same as for galectin crystals. The exposure time per crystal was 0.05 s. A total of 68 061 images were collected. The hit rate was 58.1%, from which 22 224 images were indexed in the space group *P*4_1_2_1_2 (Table 1 ▸). The final structure was refined to 2.4 Å resolution with *R*_work_ and *R*_free_ values of 17.3% and 22.1%, respectively (PDB entry 6y2n). The refined 2*m*|*F*_o_| − *D*|*F*_c_| electron density map is shown in Fig. 5 ▸(*c*).

### 3.3. Cytochrome *c* oxidase using the HVE injector   

The viscosity of the LCP phase was fine-tuned by mixing the *ba*_3_-type CcO LCP crystals with monoolein in Hamilton syringes at a crystal to monoolein ratio of 80:20. The flow rate of crystals in LCP was 1.18 µl min^−1^. The calculated exposure time per crystal was 0.028 s. A total of 214 170 images were collected with a hit rate of 4.7%, from which 6513 could be indexed in the space group *C*121. Several cycles of real-space and rigid-body refinement were performed at 3.6 Å resolution. The structure was not refined further due to the limited resolution and the fact that a room temperature high-resolution SFX structure from this crystal form has already been solved (PDB entry 5ndc). This experiment was mainly a proof of principle of SSX data collection at BioMAX using an HVE-injector. The low hit rate in this experiment was related to several beamline and software issues which are now solved.

## 4. Discussion and conclusions   

The desire to perform serial crystallography at room temperature at synchrotrons and XFELs is growing continuously, which is connected with the expanding interest of the structural biology community and the current research questions that are to be answered. The resulting demand from the user community has led to the development of beamlines dedicated to SSX. Here we have presented an initial integration of two SSX environments at BioMAX in order to collect diffraction data from micrometre-sized crystals of membrane and globular proteins at room temperature. The environments were different fixed-target scanning supports and an HVE-injector for LCP extrusion experiments. The HVE injector setup is complex, and sample preparation is arduous and time-consuming; however, it can provide data to successfully solve the structure of a membrane protein grown in LCP.

Data collection using fixed-target supports was comparatively fast, simple and required only a few microlitres of protein crystal solution, which could be grown directly on the support (Opara *et al.*, 2017 ▸). It was easier to observe microcrystals on the silicon nitride membranes than on XtalTool due to the thinner material and the absence of pores for liquid handling.

Currently, many fixed-target supports can be mounted via standard SPINE supports and soon these supports will be standardized and commercialized to perform SSX at synchrotrons. Fixed targets are a relatively cheap, fast and practical way to collect complete datasets at room temperature with minimal sample. Further advances in automated SSX data collection and data processing could help current and future users to perform serial crystallography both at synchrotrons and XFELs.

All data collections presented in this work are a result of collaborations with Swedish research groups at Gothenburg and Stockholm universities and a pharmaceutical contract research company (SARomics Biostructures). These groups are interested in applying these techniques frequently and would like to be able to utilize SSX at synchrotron beamlines as a new investigative technique. The current plans for the BioMAX SSX program also comprise the implementation of another serial crystallography environment as well – Roadrunner (Roedig *et al.*, 2017 ▸), a high-performance fixed-target scanning stage for large microfabricated crystal-holding chips. In future work the BioMAX team plan to implement SSX environments at a new beamline dedicated to serial crystallography – MicroMAX. The combination of a beam size down to 1 µm × 1 µm, together with a wide range of sample delivery systems and the possibility to perform time-resolved studies with microsecond resolution will ensure that MicroMAX will help to expand the toolbox of the Scandinavian and European SSX user community. The construction of MicroMAX has started recently and the first users will be hosted in 2022.

## Figures and Tables

**Table 1:** Data collection and refinement statistics for the proteins used Values in parentheses are for the highest resolution shell.

	Galectin-3C with lactose		R2	CcO in LCP
Sample delivery method	Fixed target (XtalTool with humidifier)	Fixed target (silicon nitride membranes)	Fixed target (silicon nitride membranes)	HVE-injector
Total amount of used sample (µl)	4	0.8	0.8	100
Flux dimension (photons s^−1^)	2 × 10^12^		2 × 10^12^	2 × 10^12^
Beam size (µm × µm)	20 × 5		20 × 5	20 × 5
Data collection temperature (K)	298		298	298
Exposure time per image (s)	0.05		0.05	0.01
Estimated dose per crystal (MGy)	0.68		0.68	0.25
No. of collected images	70537	32303	68061	214170
Average hit rate (%)	70.1	65.1	58.1	4.7
Indexing rate (%)	31.5	27.3	56.2	64.7
No. of indexed images	15555	13522	22224	6513
No. of total / unique reflections	2161463 / 15552	2762267 / 14154	12701350 / 24094	459543 / 23306
Multiplicity	139	195	527	20
Space group	*P*2_1_2_1_2_1_		*P*4_1_2_1_2	*C*121
Cell dimensions *a*, *b*, *c*, (Å)	36.6, 58.1, 63.7	35.7, 56.4, 61.7	64.3, 64.3, 153.2	145.2, 100.2, 96.6
α, β, γ (°)	90, 90, 90		90, 90, 90	90.0, 126.6, 90.0
Completeness (%)	99.9	96.3	98.6	99.7
*I*/σ(*I*)	5.66 (2.5)	5.32 (2.1)	6.73 (2.2)	2.93 (2.0)
Resolution range (Å)	60–1.70 (1.75–1.70)	61–1.70 (1.83–1.70)	59.3–2.4 (2.58–2.4)	77.5–3.6 (3.66–3.6)
*R*_split_	14.1 (37)	15.5 (50)	8.60 (64)	31.5 (53)
CC*	0.96 (0.86)	0.96 (0.63)	0.99 (0.64)	0.90 (0.59)
*R*_work_/*R*_free_ (%)	17.2 / 20.1 (30.9 / 42.0)	17.0 / 19.2 (26.9 / 29.7)	17.3 / 22.1 (23.0 / 25.9)	31.2 / 32.5 (33.4 / 38.6)
No. of atoms	1248	1244	2542	5951
Protein	1108	1149	2517	751
Water	116	72	23	N/A
Ligands	24	23	2	N/A
R.m.s.d. for bonds (Å)	0.010	0.020	0.003	0.003
R.m.s.d. for angles (°)	1.2	1.5	0.49	0.76
Average *B* value (Å^2^)	21	26	90	88
Ramachandran plot statistics				
Favoured (%)	97.8		97.7	N/A
Allowed (%)	2.2		2.32	N/A
Disallowed (%)	0		0	N/A
Rotamer outliers	0		0	N/A
PDB entry	6y4c	6y78	6y2n	N/A
